# Comparable Effectiveness of First Week Treatment with Anti-Staphylococcal Penicillin versus Cephalosporin in Methicillin-Sensitive *Staphylococcus aureus* Bacteremia: A Propensity-Score Adjusted Retrospective Study

**DOI:** 10.1371/journal.pone.0167112

**Published:** 2016-11-29

**Authors:** Erik Forsblom, Eeva Ruotsalainen, Asko Järvinen

**Affiliations:** Division of Infectious Diseases, Inflammation Center, Helsinki University Central Hospital, Finland and University of Helsinki, City of Helsinki, Finland; Aga Khan University Hospital Nairobi, KENYA

## Abstract

The objective was to compare the prognostic impact of first week treatment with anti-staphylococcal penicillin (ASP) versus cephalosporin in methicillin-sensitive *Staphylococcus aureus* bacteremia (MS-SAB). Altogether 580 patients were retrospectively followed and categorized according to first week treatment; 84% (488) received ASP (cloxacillin) and 16% (92) cephalosporin (cefuroxime or ceftriaxone). SAB management was optimized with formal bedside infectious disease specialist consultation in 88%, deep infection foci diagnosed in 77% and adjunctive rifampicin therapy given to 61% of patients. The total case fatality in 580 patients was 12% at 28 days and 18% at 90 days. When comparing effectiveness of first week ASP versus cephalosporin treatment there were no significant differences in 28-days (11% vs. 12%, OR; 1.05, 95% CI, 0.53–2.09) or 90-days (17% vs. 21% OR; 1.25, 95% CI, 0.72–2.19) outcome. In univariate analysis no prognostic impact of either first week ASP or cephalosporin treatment was observed for 28-days (OR; 0.96, 95% CI, 0.48–1.90 and OR; 1.05, 95% CI, 0.53–2.09) or 90-days (OR; 0.80, 95% CI, 0.46–1.39 and OR; 1.25, 95% CI, 0.72–2.19) outcome. Propensity-score adjusted Cox proportional regression analysis for first week treatment with cephalosporin demonstrated no significant prognostic impact at 28-days (HR 1.54, 95% CI 0.72–3.23) or 90-days (HR 1.56, 95% CI 0.88–2.86). In conclusion: There is a comparable effectiveness with respect to 28- and 90-days outcome for first week treatment with ASP versus cephalosporin in MS-SAB. The results indicate that the difference in prognostic impact between first week ASP and cephalosporin may be non-significant in patient cohorts with SAB management optimized by infectious disease specialist consultation.

## Introduction

*Staphylococcus aureus* is a leading bloodstream pathogen worldwide both in community- and healthcare-associated bacteremia (SAB) [1,2]. The prognosis of SAB is impaired by high age [2–5], hemodynamic instability [2–4] and complications like endocarditis or pneumonia [2–4] whereas deep infection focus identification [4,5] and infectious disease specialist consultation have improved outcome [2,3,5]. Despite advances in SAB management, mortality remains high, ranging from 20%-32% in recent studies [2,3]. Traditionally, anti-staphylococcal penicillin (ASP) have been the first-line choice for methicillin-sensitive *S*. *aureus* (MS-SAB) whereas cephalosporin have been regarded as a secondary alternative [6–9]. However, there are no randomized studies comparing ASP and cephalosporin in SAB and the recommendation of ASP is based on experimental observations [10,11], clinical experience and retrospective studies only [6–9]. Furthermore, the results regarding prognostic impact of ASP treatment, as compared to cephalosporin based regimens, have been controversial. In some studies, ASP has resulted in lower mortality when compared to cephalosporin [6,7,8] but a recent meta-analysis found no difference and another study showed no survival advantage with ASP over first generation cephalosporin cefazolin [9,12].

In real clinical setting treatment of SAB is often commenced with a broad spectrum antibiotic and in countries with low prevalence of methicillin-resistance cephalosporin are widely used as an empiric first-line choice in suspicion of bacteremia [7,8]. Positive blood culture results are usually received by the third day after sampling after-which empirical antimicrobial treatment may be altered into directed therapy [6,7,8]. Median antimicrobial treatment durations in previous reports have been at least 2 week [8,9]. Recent studies have demonstrated that a vast amount of deep infection foci are present already within 3 days [4] and up to 80% of SAB patients present with a deep infection focus [4,5,13]. Furthermore, meticulous deep infection focus localization and infectious disease specialist consultations are known to improved SAB prognosis [2,4,5,13]. Previous studies comparing ASP and cephalosporin in SAB have, however, not included these prognostic factors in their analyses. Moreover, the prognostic impact of continued and prolonged empiric cephalosporin treatment, as compared to targeted ASP treatment during the initial week of MS-SAB has to the best of our knowledge not been evaluated previously.

The objective of the present study was to apply propensity-score adjusted Cox proportional regression analysis to evaluate the effectiveness of first week treatment with ASP versus continued empiric cephalosporin. The study was performed in an MS-SAB patient cohort where the vast majority of patients received infectious disease specialist consultation guided SAB management and most patients had deep infection foci diagnosed.

## Materials and Methods

### Ethics statement

The trial was approved by The institutional review board of Helsinki University Central Hospital and The Ethical committee of Helsinki University Central Hospital. A written informed consent was provided by each patient.

### Patients and data collection

Adult patients with at least one positive blood culture for methicillin-sensitive *S*. *aureus* were identified. The patient cohort was assembled from two time-periods. Most SAB patients came from an earlier prospective multicenter study including all five university and seven central hospitals in Finland during January 2000 to August 2002 [4]. This cohort was further extended with all SAB cases identified retrospectively who were not included into the prospective study between years 2000 to 2002 and all SAB patients between years 2006 to 2007 from Helsinki University Central Hospital [5,14]. Two time-periods were viewed as mandatory in order to be able to exclude any unknown temporary differences in personnel or treatment practices or other factors difficult to control for. Moreover, patient data come from both written hospital archives (the earlier time-period) and electronic archives (the later time-period) and the inclusion of two time-periods enabled exclusion of differences in patient data storage patterns. We recorded the following data; age, gender, underlying diseases, acquisition of bacteremia, severe sepsis, intensive care unit (ICU) treatment and length and administration route of antibiotic therapy. Infectious diseases specialist consultations were documented. No cases of methicillin-resistant *S*. *aureus* were accepted. Exclusion criteria were: age < 18 years, pregnancy, breastfeeding, imprisonment, epilepsy, bacteremia 28 days prior to the study and poly-microbial bacteremia. Furthermore, patients with first week antibiotic treatment other than ASP or cephalosporin were excluded. Patients were followed for 90 days and primary outcome was mortality at 28- and 90 days.

### Definitions

Infectious disease specialist consultations within 7 days of positive blood cultures were recorded and categorized into formal bedside consultation or informal telephone consultation [5]. Healthcare-associated SAB was defined as bacteremia with the first positive blood culture for *S*. *aureus* obtained ≥ 48 hours after hospital admission or when the patient had remained in a long-term care facility or undergone hemodialysis within the preceding two months. McCabe’s criteria were applied to categorize underlying diseases [15]. Severe sepsis was classified as sepsis in combination with hypotension, hypo-perfusion, or organ failure [16]. Infection foci diagnosis was based on bacteriological, pathological or radiological findings or clinical suspicion only.

### Antibiotic therapy

The standard antibiotic therapy was cloxacillin, cefuroxime or ceftriaxone. First week antibiotic treatment was defined as a non-delayed onset of cloxacillin, or a continuation of empiric cefuroxime or ceftriaxone, without any interruption for at least 7 days after positive blood cultures. Length of antibiotic therapy was considered proper when administered intravenously for at least 28 days for a deep infection focus and 14 days in the absence of any deep infection. Fluoroquinolone, aminoglycoside and rifampicin served as additional antibiotic therapy. More detailed information on dosages and administration routes have been provided earlier [13,14].

### Statistical analysis

All values are provided as numbers and percentages unless otherwise specified. Pearson´s X^2^ test was applied to compare categorical variables. Odds ratios (OR) and hazard ratios (HR) with 95% confidence intervals (CI) were calculated. Univariate factors with p ≤ 0.1 were accepted for multivariate analysis. Propensity-score was calculated by logistic regression for the assignment of either ASP or cephalosporin as definitive first week antimicrobial therapy. Variables interpreted as relevant for this assignment were; gender, age > 60, healthy-nonfatal disease classification, healthcare-associated SAB, intensive care unit treatment, endocarditis and pneumonia. Next, a propensity-score adjusted Cox proportional regression model analysis was performed to estimate prognostic parameters of 28- and 90-days outcome. Kaplan-Meier interpretation with log-rank estimation was used to graphically demonstrate mortality differences. All tests were two-tailed and p<0.05 was considered as significant. Analyses were done using SPSS version 12.0 (SPSS Inc., Chicago, IL, USA).

## Results

### Patient characteristics

**Fig 1:**
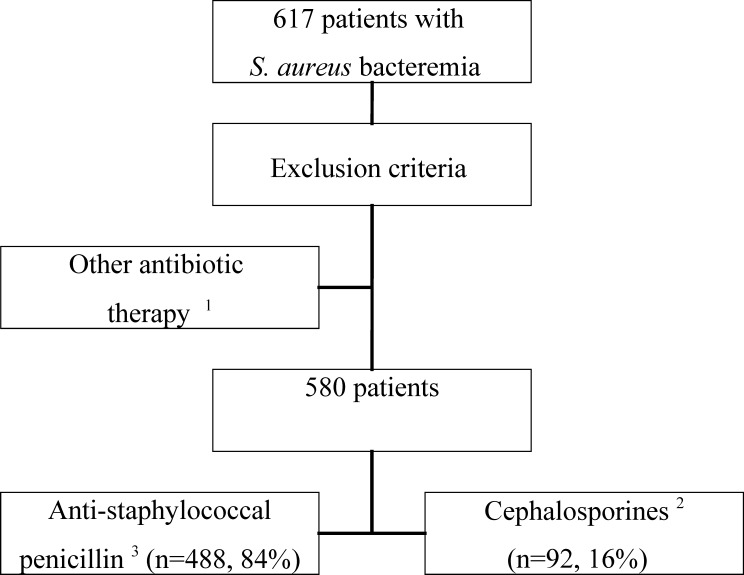
Study profile. Originally 617 patients with methicillin-sensitive *Staphylococcus aureus* bacteraemia (SAB) were identified. Patients with methicillin-resistant SAB were not included in the study. Patients were divided according to anti-staphylococcal penicillin (cloxacillin) or cephalosporin (cefuroxime or ceftriaxone) therapy during the 1^st^ week. ^1^ Carbapenemes, Clindamycin, Vancomycin or Piperasillin-Tazobactam ^2^ Cefuroxime or ceftriaxone ^3^ Cloxacillin

Altogether 617 SAB patients were identified. Exclusion criteria were accounted for and patients receiving antibiotic treatment other than ASP or cephalosporin (carbapenemes, clindamycin, vancomycin or piperasillin-tazobactam) were excluded (n = 37). In total 580 patients received either ASP or cephalosporin during the first week (Fig 1). Patients came from two time-periods; the earlier time-period (n = 467) and the later time-period (n = 113). The median time between blood culture collection and reporting of *S*. *aureus–*positive finding was three days. An intravenous antibiotic treatment effective in vitro against the *S*. *aureus* blood isolate was provided to each patient from the day of positive blood culture. Four hundred and eighty-eight (84%) patients received cloxacillin and 92 (16%) patients were treated with either cefuroxime (75, 82%) or ceftriaxone (17, 18%). No significant difference regarding gender, age, SAB acquisition, or previous hospitalizations was observed between the two groups (Table 1). Patients receiving ASP, as compared to patients treated with cephalosporin, had higher McCabe´s healthy—nonfatal classification (74% vs. 57%, p < 0.01) (Table 1). No difference with respect to severity of illness at blood culture collection time point was observed between the two groups (Table 1). Regarding additional antibiotic treatment there was no significant differences observed between the groups. Altogether 288 (50%) received fluoroquinolone, 99 (17%) had aminoglycoside whereas rifampicin was provided to 353 (61%) and only 8 (1.4%) of patients were treated with additional vancomycin therapy (Table 1).

**Table 1:** Patient characteristics, severity of illness, treatment management and outcome of 580 patients with methicillin-sensitive *Staphylococcus aureus* bacteraemia (SAB). Patients are categorised according to first week antimicrobial therapy into anti-staphylococcal penicillin or cephalosporin. Values are n (%). NS = non-significant.

	Antibiotic therapy		Cephalosporin vs. Anti-staphylococcal penicillin	
Variables	Anti-staphylococcal penicillin ^1^ n = 488 (84)	Cephalosporin ^2^ n = 92 (16)	OR (95% CI)	p- value
**Patient characteristics**				
Male sex	302 (62)	58 (63)	1.05 (0.66–1.66)	NS
Age > 60 years	238 (49)	35 (38)	0.65 (0.41–1.02)	NS
Healthcare-associated SAB	258 (53)	49 (53)	1.02 (0.65–1.59)	NS
Healthy-nonfatal disease ^**A**^	362 (74)	52 (57)	0.45 (0.29–0.72)	<0.01
Previous hospitalization ^**B**^	259 (53)	55 (60)	1.31 (0.84–2.07)	NS
Intensive care unit ^**C**^	89 (18)	20 (22)	1.25 (0.72–2.15)	NS
Deep infection focus	383 (78)	64 (70)	0.63 (0.38–1.03)	NS
Endocarditis	81 (17)	9 (10)	0.55 (0.26–1.13)	NS
Pneumonia	181 (37)	37 (40)	1.14 (0.72–1.79)	NS
Vancomycin	8 (2)	0	—	—
Fluoroquinolone	245 (50)	43 (47)	0.87 (0.56–1.36)	NS
Aminoglycoside	79 (16)	20 (22)	1.44 (0.83–2.49)	NS
Rifampicin	301 (62)	52 (57)	0.81 (0.52–1.27)	NS
**Mortality at 28 days**	56 (11)	11 (12)	1.05 (0.53–2.09)	NS
**Mortality at 90 days**	84 (17)	19 (21)	1.25 (0.72–2.19)	NS

^1^ Cloxacillin

^2^ Cefuroxime or ceftriaxone

^A^ Classification according to McCabe and Jackson [15]

^B^ Within 2 months prior to blood culture collection

^C^ Severity of illness at blood culture collection time point

### Deep infection foci

Altogether 447 (77%) of patients had at least one deep infection focus diagnosed (Table 1). There were no differences in patients receiving ASP or cephalosporin during the first week with respect to occurrence of deep infection foci, endocarditis or pneumonia (Table 1).

### Infectious disease specialist consultation

Infectious disease specialist consultation guided SAB management was provided to almost all patients 565 (97%) whereas 15 (3%) were managed without any consultation. The consultations were mostly formal bedside 511 (88%) whereas 54 (9%) had informal telephone consultations (Table 2).

**Table 2:** Univariate analysis of prognostic factors for 28- and 90-days mortality in 580 patients with methicillin-sensitive *Staphylococcus aureus* bacteremia. Values are expressed as N (%). NS = non-significant.

	28-days				90-days			
Variables	Died 67 (12)	Survived 513 (88)	OR (95% CI)	p- value	Died 103 (18)	Survived 477 (82)	OR (95% CI)	p- value
**Patient characteristics**								
Male sex	43 (64)	317 (62)	1.11 (0.65–1.88)	NS	62 (60)	298 (62)	0.91 (0.59–1.41)	NS
Age > 60 years	52 (78)	221 (43)	4.58 (2.51–8.35)	< 0.001	73 (71)	200 (42)	3.37 (2.12–5.35)	< 0.001
Healthcare-associated SAB	36 (54)	271 (53)	1.04 (0.62–1.73)	NS	65 (63)	242 (51)	1.66 (1.07–2.58)	< 0.05
Healthy—nonfatal disease ^**A**^	28 (42)	386 (75)	0.24 (0.14–0.40)	< 0.001	38 (37)	376 (79)	0.16 (0.10–0.25)	< 0.001
Previous hospitalization ^**B**^	36 (54)	278 (54)	0.98 (0.59–1.64)	NS	64 (62)	250 (52)	1.49 (0.96–2.31)	NS
Intensive care unit ^**C**^	23 (34)	86 (17)	2.59 (1.49–4.52)	< 0.01	29 (28)	80 (17)	1.95 (1.19–3.18)	< 0.01
Endocarditis	19 (28)	71 (14)	2.46 (1.37–4.43)	< 0.01	26 (25)	64 (13)	2.18 (1.30–3.65)	< 0.01
Pneumonia	41 (61)	177 (35)	2.99 (1.77–5.06)	< 0.001	63 (61)	155 (32)	3.27 (2.11–5.08)	< 0.001
Cephalosporin ^**D**^	11 (16)	81 (16)	1.05 (0.53–2.09)	NS	19 (18)	73 (15)	1.25 (0.72–2.19)	NS
Anti-staphylococcal penicillin ^**E**^	56 (84)	432 (84)	0.96 (0.48–1.90)	NS	84 (82)	404 (85)	0.80 (0.46–1.39)	NS
Rifampicin therapy	14 (21)	238 (46)	0.31 (0.17–0.56)	< 0.001	30 (29)	252 (53)	0.37 (0.23–0.58)	< 0.01
Fluoroquinolone	31 (46)	257 (50)	0.86 (0.52–1.43)	NS	48 (47)	240 (50)	0.86 (0.56–1.32)	NS
Aminoglycoside	13 (19)	86 (17)	1.19 (0.63–2.29)	NS	20 (19)	79 (17)	1.21 (0.70–2.09)	NS
Formal IDSC ^**F**^	58 (87)	453 (88)	0.85 (0.40–1.81)	NS	82 (80)	429 (90)	0.44 (0.25–0.77)	< 0.01

^A^ Classification according to McCabe [15]

^B^ Within 2 months prior to blood culture collection

^C^ At positive blood culture time point

^D^ Cephalosporin i.e. Cefuroxime or ceftriaxone

^E^ Anti-staphylococcal penicillin i.e. cloxacillin

^F^ Formal bedside infectious disease specialist consultation

### Mortality

**Fig 2:**
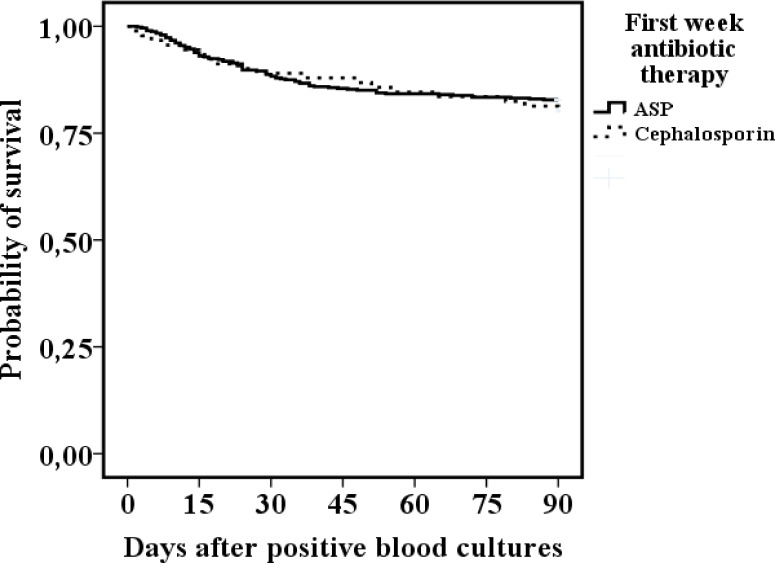
Kaplan-Meier interpretation of 90 days outcome in 580 methicillin-sensitive *Staphylococcus aureus* bacteremia patients receiving either anti-staphylococcal penicillin (ASP, cloxacillin) (n = 488) or cephalosporin (cefuroxime or ceftriaxone) (n = 92) as first week antibiotic treatment after positive blood cultures. Log-rank non-significant.

The total case fatality in 580 patients was 12% at 28 days and 18% at 90 days. There was no difference in mortality between first week ASP and cephalosporin treatments at 28 days (11% vs. 12%, OR; 1.05) or at 90 days (17% vs. 21%, OR; 1.25) (Tables 1 and 2, Fig 2).

In univariate analysis; parameters with 28-days prognostic impact were: age > 60 (OR; 4.58, p < 0.001), healthy–nonfatal underlying disease (OR; 0.24, p < 0.001), intensive care unit (OR; 2.59, p < 0.01), endocarditis (OR; 2.46, p < 0.01), pneumonia (OR; 2.99, p < 0.001) and rifampicin therapy (OR; 0.31, p <0.001) whereas parameters for 90-days prognostic impact were very similar to those of 28-days with the additional parameters of healthcare-associated bacteremia (OR; 1.66, p < 0.05) and formal bedside infectious disease consultation (OR; 0.44, p < 0.01) (Table 2). At both 28- and 90-days neither ASP (OR; 0.96, 95% CI, 0.48–1.90 and OR; 0.80, 95% CI, 0.46–1.39) nor cephalosporin treatment (OR; 1.05, 95% CI, 0.53–2.09 and OR; 1.25, 95% CI, 0.72–2.19) had any prognostic impact (Table 2).

A Kaplan-Meier interpretation of the differences in outcome between first weeks ASP versus cephalosporin treatment produced a non-significant log-rank comparison (Fig 2).

Propensity-score adjusted Cox proportional regression analysis for first week treatment with cephalosporin demonstrated no significant prognostic impact at 28-days (HR 1.54; 95% CI 0.72–3.23) or at 90-days (HR; 1.56, 95% CI 0.88–2.86) (Tables 3 and 4). Parameters with prognostic impact in propensity-score adjusted Cox proportional regression analysis for 28-days outcome were intensive care unit treatment (HR; 2.60, p < 0.01), pneumonia (HR; 2.29, p < 0.05) and additional therapy with rifampicin (HR; 0.24, p < 0.01), whereas parameters for 90-days outcome were; age > 60 years (HR; 5.15, p < 0.01), endocarditis (HR; 4.74, p < 0.01), pneumonia (HR; 2.18, p < 0.01), formal bedside infectious disease specialist consultation (HR; 0.39, p < 0.05) and additional therapy with rifampicin (HR; 0.34, p < 0.01).

**Table 3:** Propensity-score adjusted Cox proportional regression analysis for 28-days mortality among 580 patients with methicillin-sensitive *Staphylococcus* aureus bacteremia receiving either anti-staphylococcal penicillin (n = 92) or cephalosporin (n = 488) as first week antimicrobial treatment.

	PS-adjusted multivariate HR (95% CI)	p- value
Anti-staphylococcal penicillin ^**1**^	1.0	---
Cephalosporin ^**2**^	1.54 (0.72–3.23)	NS
Male sex	1.26 (0.72–2.21)	NS
Age > 60 years	3.46 (0.83–14.4)	NS
Healthy-nonfatal disease ^**A**^	0.30 (0.05–1.89)	NS
Healthcare-associated bacteremia	0.65 (0.34–1.24)	NS
Intensive care unit ^**B**^	2.60 (1.34–5.03)	< 0.01
Endocarditis	2.26 (0.65–7.88)	NS
Pneumonia	2.29 (1.31–4.01)	< 0.05
Formal IDSC ^**C**^	1.03 (0.24–4.50)	NS
Rifampicin therapy ^**D**^	0.24 (0.13–0.49)	< 0.01

^1^ Cloxacillin

^2^ Cefuroxime or ceftriaxone

^A^ Classification according to McCabe and Jackson [15]

^B^ At blood culture collection time-point

^C^ Infectious disease specialist consultation

^D^ Additional antibiotic therapy

**Table 4:** Propensity-score adjusted Cox proportional regression analysis for 90-day mortality among 580 patients with methicillin-sensitive *Staphylococcus* aureus bacteremia receiving either anti-staphylococcal penicillin (n = 92) or cephalosporin (n = 488) as first week antimicrobial treatment.

	PS-adjusted multivariate HR (95% CI)	P- value
Anti-staphylococcal penicillin ^**1**^	1.0	---
Cephalosporin ^**2**^	1.56 (0.88–2.86)	NS
Male sex	1.15 (9.74–1.78)	NS
Age > 60 years	5.15 (1.77–15.0)	< 0.01
Healthy-nonfatal disease ^**A**^	0.75 (0.20–2.81)	NS
Healthcare-associated bacteremia	1.29 (0.78–2.13)	NS
Intensive care unit ^**B**^	1.35 (0.79–2.31)	NS
Endocarditis	4.74 (1.94–11.6)	< 0.01
Pneumonia	2.18 (1.39–3.42)	< 0.01
Formal IDSC ^**C**^	0.39 (0.16–0.99)	< 0.05
Rifampicin therapy ^**D**^	0.34 (0.22–0.53)	< 0.01

^1^ Cloxacillin

^2^ Cefuroxime or ceftriaxone

^A^ Classification according to McCabe and Jackson [15]

^B^ At blood culture collection time-point

^C^ Infectious disease specialist consultation

^D^ Additional antibiotic therapy

The patient cohort came from two separate time-periods. This raises the question whether changes in clinical practice over the years have favored one or the other treatment group. As a further analysis the propensity-score adjusted Cox proportional regression analysis was performed separately for the larger patient subgroup from the earlier time-period. However, the results remained similar to those of Tables 3 and 4 with no significant prognostic impact for first week treatment with cephalosporin at 28-days (HR; 1.91, 95% CI 0.45–6.13) and at 90-days (HR; 1.85, 95% CI 0.74–4.65).

The cephalosporin group contained both cefuroxime and ceftriaxone i.e. regimens from two different cephalosporin generations. As a further investigation the main analyses with propensity-score adjusted Cox proportional regression analysis were re-performed by excluding ceftriaxone and comparing only first week treatment with ASP versus cefuroxime. However, the results did not deviate from those of Tables 3 and 4 with no significant prognostic impact for first week treatment with cefuroxime at 28-days (HR; 1.78, 95% CI 0.77–4.12) or at 90-days (HR; 1.71, 95% CI 0.90–3.26).

To further evaluate the stability of the main results, patients were categorized in turn according to age or underlying conditions, i.e. two major factors that may have strong influence on outcome, and the propensity-score adjusted Cox proportional regression analysis for 90-days prognostic parameters re-performed. However, when including only patients aged < 60 years or aged > 60 years the 90-days prognostic impact for first week treatment with cephalosporin was non-significant (HR; 2.70, 95% CI 0.85–6.47 and HR; 1.10, 95% CI 0.52–2.35, respectively). Further, when including only patients with McCabe´s healthy-nonfatal or McCabe´s ultimately-rapidly fatal classification the 90-days prognostic impact for first week treatment with cephalosporin was non-significant as well (HR; 0.79, 95% CI 0.26–2.37 and HR; 1.64, 95% CI 0.79–4.35, respectively).

## Discussion

The main finding of the present study was a comparable effectiveness with respect to 28- and 90-days outcome for first week treatment with ASP versus cephalosporin in MS-SAB. The results were achieved by propensity-score adjusted Cox proportional regression analysis and the observations remained after re-performing the analysis by categorization of the patient cohort according to data collection time-period, age and underlying conditions.

Several retrospective studies [6,7,8,9,12,17] have compared the prognostic impact of ASP, mostly cloxacillin, dicloxacillin or oxacillin [6,8,17], and cephalosporin, mostly cefuroxime [6–8]. The patient cohorts have included MS-SAB [7–9,12,17] whereas one study included penicillin-sensitive SAB (PS-SAB) patients only [6]. These studies demonstrated lower [6–8] or indifferent [9,17] mortalities at 30 and 90 days for ASP as compared to cephalosporin. Two studies identified cefuroxime treatment as an independent parameter for higher 30- and 90-days mortality as compared to penicillin for PS-SAB or dicloxacillin for MS-SAB [6,8]. Furthermore, empirical onset of cloxacillin or cefazolin, as compared to cefuroxime, ceftriaxone or cefotaxime were associated to a significantly lower 30-days mortality in one report [7]. In contrast, similar clinical cure rates for oxacillin and cefazolin have been reported [17]. A recent meta-analysis found no significant difference in 30 or 90 days outcome for ASP versus cephalosporin therapy in MS-SAB [12].

The comparison of results in the studies mentioned above is challenging due to variations in clinical settings. Previous studies include a wide range of different ASP agents i.e. dicloxacillin [6,8], cloxacillin [7,17], nafcillin [9] and cephalosporin from different generations i.e. cefazolin [7,9,17], cefuroxime [6,7,8] or ceftriaxone [7]. The present study applied the ASP agent cloxacillin which was used in only two earlier report [7,17]. As with the present study, many previous reports have altered empiric antimicrobial treatment into directed treatment within 3 days of blood culture collection [6,7,8]. However, durations of directed antimicrobial treatment in earlier reports differ with some authors reporting median treatment durations of 15–17 days [8,9]. The present study regarded length of antibiotic therapy as proper when administered intravenously for at least 28 days for a deep infection focus and 14 days in the absence of any deep infection.

Previous studies on the impact of ASP and cephalosporin in SAB do not include or do not comment on the presence of any infectious disease specialist consultation [6,7,8,9,12,17] whereas in the present study altogether 88% received formal bedside infectious disease specialist consultation. Moreover, previous studies have not evaluated the potential positive prognostic impact of adjunctive rifampicin treatment whereas in the present study 61% of patients were provided with rifampicin treatment [6,7,8,9,12,17]. Previous studies have reported occurrence of secondary metastatic (deep) infection foci in 11%-21% of patients [6,8,17] with pneumonia in 7%-17% and endocarditis in 6%-10% [6,7,8,17] of patients. In the present study 77% of patients were diagnosed with a deep infection focus including 16% endocarditis and 38% pneumonia and most deep foci were diagnosed within 3 days of positive blood cultures [4]. Infectious disease specialist consultations, meticulous deep infection foci localization and adjunctive rifampicin treatment have previously been associated to enhanced SAB prognosis [2,3,4,5,14]. However, several of the parameters with prognostic impact in the present study have been reported earlier i.e. age, underlying conditions, intensive care unit treatment, diagnosis of endocarditis and pneumonia [2–5,7–9], infectious disease specialist consultation [2,3,5] and adjunctive rifampicin therapy [14].

The results of the present study diverge from reports stating that ASP, as compared to cephalosporin, associates to significantly lower mortality rates [6–8] and studies presenting cefuroxime as an independent parameter for 30 or 90 days mortality [6,8]. Altogether, previous studies on ASP and cephalosporin in MS-SAB report overall mortality rates of 10–37% at 30-days [6–9] and 27–49% at 90-days [7,8,17]. These mortality figures are considerable higher than those of the present study with 12% at 28 days and 18% at 90 days. The lower mortality in this study may have two explanations. First, we might have missed some of the more severe cases. Second, more importantly, factors such as non-delayed onset of an intravenous antibiotic treatment effective in vitro against the *S*. *aureus* blood isolate provided to each patient from the day of positive blood culture, proper duration of antibiotic therapy, infectious disease specialist consultation guided SAB management, meticulous deep infection foci localization and adjunctive rifampicin therapy have contributed to lower mortality rates. These prognostic factors have previously have been linked to favorable SAB outcomes [2,3,4,5,14].

There are weaknesses in the present study which have to be accounted for when interpreting results.

First, the cephalosporin group included both second (cefuroxime) and third (ceftriaxone) generation cephalosporin. Analyses with combination of antibiotic regimens from different cephalosporin generations may be viewed as controversial. However, in the Finnish healthcare system cefuroxime and ceftriaxone are commonly applied empiric antibiotics among infectious diseases. This motivates the analyzing of these two together. However, to further investigate the stability of the results, the main analyses with propensity-score adjusted Cox proportional regression analysis were re-performed by excluding ceftriaxone and comparing only first week treatment with ASP versus cefuroxime. The results of this closely resembled the main results with no significant prognostic difference at 28- or 90-days between the ASP and cefuroxime treatment.

Second, the patient cohort came from two separate time-periods. The two different time-periods were viewed as mandatory in order to be able to account for any changes in personnel or clinical treatment practices. However, two time-periods may raise the question whether changes in clinical practice have favored one or the other treatment group. To further analysis this, the propensity-score adjusted Cox proportional regression analysis was performed separately for the larger patient subgroup from the earlier time-period and the results demonstrated no significant prognostic difference at 28- or 90-days between the ASP and cefuroxime treatment.

Third, retrospective studies may be biased by differences in the patient groups such as age and underlying conditions. Moreover, it is well known that severely ill patients, as compared to patients with a more optimistic clinical condition, are more likely to receive broader spectrum antimicrobial therapy. This ´´*confounding by indication”* may further bias retrospective cohort analyses [18]. However, potential bias may be reduced through propensity-score adjusted analyses and through categorization of data according to various parameters [19]. In the present study patients receiving ASP had significantly less severe underlying conditions according to McCabe´s classification. Further, although statistically not significant, there were minor differences with respect to gender, age, bacteremia acquisition and need for intensive care unit treatment i.e. factors that may have influenced the final results. Moreover, the difference in patient number between ASP (n = 488) and cephalosporin (n = 92) may give suspicion that confounders of prognostic importance might have gone unaccounted for. However, the main observations were achieved by propensity-score adjusted analysis, correcting for potential differences between the ASP and cephalosporin group. Moreover, sub-analyses with categorization according to age and underlying conditions produced results that closely resembled the main observations. This indicates that the main results are robust and makes the risk for statistical bias low.

Fourth, with respect to the earlier time-period, the question of whether the data in the present study is valid to current clinical practice may be raised. However, although the management of SAB is continuously developed as new clinical research is published, there are fundamental elements of SAB management that has remained unchanged over the years such as prompt onset and correct duration of antibiotic treatment and meticulous identification and eradication of deep infection foci [1]. Infectious disease specialist consultation is known to ensure highly optimized SAB management [2,5]. The authors view that the high presence (88%) of formal bedside infectious disease specialist consultation in the present study has both ensured recording of relevant clinical patient information and guaranteed high standard clinical management of SAB. Hence, the patient data of the present study is not outdated for current clinical practice.

In conclusion, we observed a comparable effectiveness with respect to 28- and 90-days outcome for first week treatment with ASP versus cephalosporin in MS-SAB. The results indicate that the difference in prognostic impact between first week ASP and cephalosporin may be non-significant in patient cohorts with SAB management optimized by infectious disease specialist consultation.
